# Fetal and maternal outcome in patients with active lupus nephritis: comparison between new-onset and pre-existing lupus nephritis

**DOI:** 10.1186/s12882-021-02633-2

**Published:** 2021-12-21

**Authors:** Xing-Ji Lian, Li Fan, Xi Xia, Xia-Min Huang, Hong-Jian Ye, Xue-Qing Yu, Hai-Tian Chen, Wei Chen

**Affiliations:** 1 Department of Nephrology, The First Affiliated Hospital, NHC Key Laboratory of Nephrology, Guangdong Provincial Key Laboratory of Nephrology, Sun Yat-sen University, No. 58 Zhongshan Er Lu, Guangzhou, 510080 China; 2 Division of Nephrology, Guangdong Provincial People’s Hospital and Guangdong Academy of Medical Sciences, Guangzhou, 510080 China; 3 Department of Obstetrics and Gynecology, The First Affiliated Hospital of Sun Yat-sen University, No. 58 Zhongshan Er Lu, Guangzhou, 510080 Guangdong China

**Keywords:** lupus nephritis, glomerulonephritis, kidney disease outcome, pregnancy outcome, new-onset

## Abstract

**Background:**

This study aimed to investigate fetal and maternal outcomes in women with active lupus nephritis (LN). Specifically, we compared women who had new-onset LN and those with pre-existing LN during pregnancy.

**Methods:**

Patients with active LN during pregnancy were divided into the new-onset group (LN first occurred during pregnancy) and the pre-existing group (a history of LN) on the basis of the onset time of LN. Data on clinical features, laboratory findings, and pregnancy outcome were collected and analyzed between the two groups. Multivariate logistic regression analysis was used to compare the effects of active LN on adverse pregnancy outcomes.

**Results:**

We studied 73 pregnancies in 69 women between 2010 and 2019. Of these, 38 pregnancies were in the pre-existing LN group and 35 were in the new-onset group. Patients with pre-existing LN had a higher risk of composite adverse fetal outcomes than those with new-onset LN [adjusted odds ratio (ORs), 44.59; 95% confidence interval (CI), 1.21–1664.82; *P* = 0.039]. However, the two groups had similar adverse maternal outcomes (ORs, 1.24; 95% CI, 0.36–4.29). Serum albumin and proteinuria significantly improved after pregnancy (*P* < 0.001). Kaplan–Meier analysis showed that the long-term renal outcome was similar between the two groups.

**Conclusions:**

Pregnant patients with pre-existing LN were associated with a higher risk of composite adverse fetal outcomes than those with new-onset LN. However, these two groups of patients had similar adverse maternal outcomes. The long-term renal outcomes were not different after pregnancy between these two groups.

**Supplementary Information:**

The online version contains supplementary material available at 10.1186/s12882-021-02633-2.

## Introduction

Lupus nephritis (LN), which is the most common form of secondary glomerulonephritis in China, is a major contributor to morbidity and mortality of systemic lupus erythematosus (SLE) [1, 2]. LN most frequently affects female patients of child-bearing age [3, 4]. This high-risk group can be separated into two subsets of patients who already have a long history of LN at the time of conception and patients with LN newly occurring after conception. Currently, patients with LN can consider pregnancy if they are in remission for more than 6 months after induction of treatment [5]. However, active kidney disease (new-onset or pre-existing) can occur during pregnancy, which poses a great challenge to clinicians and raises concern [6].

Most previous studies focused on fetal and maternal outcomes in patients with stable/mildly active SLE [7–9]. Moreover, some studies evaluated the clinical features between new-onset and pre-existing SLE during pregnancy and its effects on adverse pregnancy outcomes [10–12]. These studies showed clinically relevant differentiation due to the significant effect of organ involvement. Nevertheless, studies that investigated patients with active LN, particularly studies on those with new-onset LN during pregnancy and puerperium, are limited. Therefore, this study aimed to investigate adverse pregnancy outcomes and the long-term renal outcome between women with active LN with new-onset and pre-existing LN.

## Methods

### Study design and population

All patients with a diagnosis of active LN who were older than 18 years and visited the First Affiliated Hospital of Sun Yat-sen University from December 2010 to December 2019 for at least one pregnancy were enrolled in this observational study. Each patient fulfilled the diagnostic criteria for SLE that were revised in 1997 by the American College of Rheumatology. The study exclusion criteria were as follows: (1) no active LN; (2) no singleton intrauterine pregnancy confirmed by ultrasound; (3) drug-induced SLE and a malignant tumor; and (4) absence of data of pregnancy and delivery. On the basis of renal disease, patients with active LN were divided into the pre-existing group (pregnant patients with a history of LN) and the new-onset group (patients newly diagnosed with LN during pregnancy). The diagnosis of SLE or LN concurred with the disease onset in the new-onset group. Active LN was defined as proteinuria > 0.5 g/24 h, active urinary sediment (> 3 red blood cells/high-power field, > 5 white blood cells/high-power field, or cellular casts), or estimated creatinine clearance < 60 ml/min/1.73 m^2^ with active urinary sediment [13]. All patients with LN in the pre-existing and new-onset group had active LN during pregnancy based upon the criteria defined. The study protocol conformed to the provisions of the Declaration of Helsinki (as revised in 2013). The Ethics Committee of the First Affiliated Hospital of Sun Yat-sen University reviewed and approved the study protocol. Individual written informed consent for this retrospective analysis was waived.

### Data collection and definitions

We collected data on women with LN at the time of perinatal care and delivery. Blood samples for a complete blood count, blood biochemical test, complement C3 and C4 levels, antibodies [e.g., anti-nuclear antibody (ANA), anti-double-stranded DNA (dsDNA) antibody, antiphospholipid antibody, anti-ribosomal ribonucleoprotein (rRNP) antibody, anti-Sjögren syndrome antigen A (SSA) and anti-Sjögren syndrome antigen B antibody (SSB)] were obtained. Urinary sediment analysis and 24-h urine protein levels were recorded. We also analyzed information on the past gestational history, gestational weeks, delivery mode, and neonatal birth weight. LN and obstetric complications were evaluated by daily progress notes and medical records at discharge. When evaluating the long-term renal outcomes of women who gave birth, we excluded patients with end-stage renal disease or those who received renal replacement therapy during pregnancy, as well as those who lacked complete records on long-term renal outcomes. The estimated glomerular filtration rate (eGFR) was measured by the Chronic Kidney Disease Epidemiology Collaboration creatinine formula [14].

### Definition of adverse pregnancy outcomes and long-term renal outcomes

Adverse pregnancy outcomes included adverse fetal and maternal outcomes. Adverse fetal outcomes included the following: (1) fetal loss, including spontaneous abortions (before the 20th week of gestation caused by natural factors), stillbirth (intrauterine fetal demise after the 20th week of gestation), and therapeutic abortions (artificial termination of pregnancy due to uncontrolled diseases, obstetric complications, or influence of drugs); (2) preterm birth, which was defined as delivery < 37 weeks of gestation; (3) low birth weight, which was defined as a birth weight < 2.5 kg; and (4) fetal distress referred to as fetal hypoxia and acidosis, which could pose a risk to the health of the fetus. More specific characteristics and definitions have been previously described [15].

Adverse maternal outcomes included preeclampsia (new-onset hypertension and proteinuria > 300 mg/day after 20 weeks’ gestation) and severe preeclampsia (new onset of systolic blood pressure > 160 mm Hg or diastolic blood pressure > 110 mm Hg with severe proteinuria > 5 g/d), and cesarean delivery. A composite of adverse maternal or fetal outcomes was separately defined as one in which the composition of the above-mentioned adverse pregnancy outcomes occurred for a single pregnancy. Long-term renal outcomes included complete or partial renal remission. Complete renal remission was defined as serum creatinine levels < 1.0 mg/dl and proteinuria < 500 mg/24 h. Partial renal remission was defined as serum creatinine levels < 1.0 mg/dl, and proteinuria was 500–1000 mg/d or random uPCR 0.5–1.0 or dipstick < 2+ [16, 17]..

### Statistical analysis

Continuous variables are shown as the mean ± standard deviation or median (25th, 75th percentiles), and were analyzed by the Student’s t-test or Wilcoxon rank-sum test. Categorical variables are described as percentage and frequency, and were compared by Pearson’s chi-square test or Fisher’s exact test. A multivariable logistic regression model was used to evaluate the effect of LN on adverse pregnancy outcomes after adjustment for maternal characteristics. Renal function-related indices at the time of and after pregnancy were compared using the paired t or Wilcoxon rank-sum test. Survival curves were performed by the Kaplan–Meier estimate and analyzed using the log-rank test. The adjusted odds ratios (ORs) with 95% confidence interval (CI) are reported. All statistical tests were two sided and *P* values < 0.05 were considered significant. Statistical analysis was conducted by Stata version 15 (Stata Corp LP, College Station, TX, USA).

## Results

### Clinical characteristics at the time of delivery between the two groups

A total of 69 LN women with 73 pregnancies were included in this study, of which 38 pregnancies had a history of LN (pre-existing LN group) and 35 had new-onset LN during pregnancy (new-onset LN group) (Supplementary Fig. 1). At the time of delivery, patients in the new-onset LN group were 3 years younger than those in the pre-existing LN group. Body mass index and blood pressure were similar between the two groups. One third of the total patients with LN had a history of an adverse pregnancy with no difference between two groups. Patients in the pre-existing LN group received more aggressive treatment (26.3%) with steroids and immunosuppressive drugs) compared with those in the new-onset LN group (2.9%, *P* = 0.007), but they received similar treatment post-pregnancy. The proteinuria, eGFR and serum albumin levels were not different between LN patients with pre-existing LN and new onset LN (Table 1).

**Table 1:** Clinical characteristics at the time of delivery

Variables	Total (n = 73)	Pre-existing LN (n = 38)	New-onset LN (n = 35)	*P* value
Age, years	28.0 (25.0, 31.0)	29.0 (26.0, 32.0)	26.0 (23.0, 29.5)	0.011
Body mass index, kg/m^2^	25.2 (23.4, 26.6)	24.7 (23.7, 27.9)	25.4 (23.3, 26.0)	0.71
Systolic blood pressure, mmHg	126.0 (112.0, 139.0)	129.0 (119.5, 142.0)	124.0 (110.0, 138.0)	0.25
Diastolic blood pressure, mmHg	80.0 (70.0, 90.0)	81.5 (71.5, 90.5)	75.5 (69.5, 92.5)	0.38
Mean arterial pressure, mmHg	94.3 (83.7, 106.7)	98.8 (87.8, 107.7)	90.7 (83.5, 107.8)	0.34
**History of pregnancy**				
First-time pregnancy (%)	33 (45.2)	17 (44.7)	16 (45.7)	0.82
Adverse pregnancy history (%)	20 (27.4)	12 (31.63)	8 (22.9)	0.62
Duration of SLE diagnosis to pregnancy, years	**-**	7 (4.0, 10.0)	-	-
Duration of LN diagnosis to pregnancy, years	**-**	6 (2.0, 9.3)	-	-
**Treatment during pregnancy (%)**				
Prednisone and immunosuppressant	11 (15.1)	10 (26.3)	1 (2.9)	0.007
Prednisone alone	65 (89.0)	35 (92.1)	30 (85.7)	0.28
**Treatment post pregnancy (%)**				
Prednisone and cyclophosphamide	20 (27.4)	9 (23.7)	11 (31.4)	0.40
Prednisone and methotrexate	5 (6.8)	1 (2.6)	4 (11.4)	0.18
Prednisone and calcineurin inhibitors	9 (12.3)	6 (15.8)	3 (8.6)	0.48
Prednisone and mycophenolate mofetil	8 (11.1)	4 (10.5)	4 (11.4)	0.86
**Laboratory Values**				
Hemoglobin, g/L	91.0 (76.0, 108.0)	98.0 (81.0, 112.0)	85.0 (75.5, 103.5)	0.12
Platelet, ×10^9^/L	179.0 (134.0, 257.0)	203.0 (137.0, 260.0)	173.0 (123.0, 239.0)	0.63
White blood cell, ×10^9^/L	7.8 (5.5, 9.9)	8.8 (7.0, 10.8)	5.8 (4.3, 9.3)	0.012
Urea nitrogen, mg/dL	35.5 (21.0, 54.1)	34.9 (22.8, 55.9)	35.5 (19.8, 47.5)	0.64
Serum creatinine, mg/dL	0.8 (0.6, 1.1)	0.7 (0.6, 1.2)	0.8 (0.6, 1.1)	0.51
eGFR, mL/min/1.73 m^2^	106.0 (67.4, 124.9)	103.6 (63.6, 125.1)	108.5 (71.2, 124.9)	0.79
Serum albumin, g/dL	2.5 (2.1, 2.9)	2.3 (1.9, 2.9)	2.5 (2.2, 2.7)	0.44
Proteinuria, g/day	3.8 (2.0, 6.0)	4.5 (2.8, 5.1)	3.8 (2.0, 6.5)	0.93
> 3 red blood cells / high-power field (%)	49 (67.1)	24 (63.2)	25 (71.4)	0.29
> 5 white blood cells/ high-power field (%)	46 (63.0)	21 (55.3)	25 (71.4)	0.25

Data are presented as median (range) or as number (percentage). The p-values of categorical variable obtained with the Chi square or Fisher's exact test and continuous variables obtained with the Mann Whitney U-test.

*LN* lupus nephritis, *SLE* systemic lupus erythematosus, *eGFR* estimated glomerular filtration rate

### Immunological parameters, organ involvement, and complications between the two groups

There were no significant differences in the erythrocyte sedimentation rate and positive rates of autoimmune antibodies, including anti-dsDNA antibody, ANA, anti-SSA antibody, and anti-SSB antibody, between the two groups. However, hypocomplementemia, positivity for anti-rRNP and antiphospholipid antibody were significantly more likely to occur in patients with new-onset LN than in those with pre-existing LN (all *P* value < 0.05) (Table 2). The involvement of the kidney and other organs, as well as complications, were not significantly different between the two groups (Supplementary Table 1). Three of the seven patients with dialysis therapy had acute kidney injury, while other four patients suffered from chronic renal failure and required dialysis therapy after discharge.

**Table 2:** Immunological parameters at the time of delivery

Variables	Total (n = 73)	Pre-existing LN (n = 38)	New-onset LN (n = 35)	*P* value
ESR, mm/h	37.0 (18.0, 47.0)	40.0 (18.0, 49.0)	34.0 (23.0, 43.0)	0.41
Anti-dsDNA antibodies, U/ml	4.5 (2.6, 83.0)	5.5 (2.3, 95.0)	4.4 (2.7, 20.0)	0.64
Anti-dsDNA antibody positive (%)	65 (89.0)	32 (84.2)	33 (94.3)	0.41
ANA positive (%)	69 (94.5)	34 (89.5)	35 (100)	0.16
Antiphospholipid antibody positivity (%) *	15 (20.5)	4 (10.5)	11 (31.4)	0.027
Anti-cardiolipin IgG positivity (%)	8 (11.0)	2 (5.3)	6 (17.1)	0.14
Anti-cardiolipin IgM positivity (%)	7 (9.6)	1 (2.6)	6 (17.1)	0.035
β2 glycoproteins positive, n (%)	5 (6.8)	1 (2.6)	4 (11.4)	0.19
Anti-SSA antibody positive (%)	40 (54.8)	16 (42.1)	24 (68.6)	0.11
Anti-SSB antibody positive (%)	12 (16.4)	4 (10.5)	8 (22.9)	0.34
Anti-Sm antibody positive (%)	19 (26.0)	6 (15.8)	13 (37.1)	0.10
Anti-rRNP antibody positive (%)	31 (42.5)	11 (28.9)	20 (57.1)	0.04
Complement C3, mg/dl	0.5 (0.4 0.8)	0.6 (0.5, 0.9)	0.4 (0.3, 0.6)	< 0.01
Complement C4, mg/dl	0.1 (0.1, 0.1)	0.1 (0.1, 0.2)	0.1 (0.1, 0.1)	0.02
Low complement C3 (%)	54 (74.0)	23 (60.5)	31 (88.6)	0.006
Low complement C4 (%)	55 (75.3)	23 (60.5)	32 (91.4)	0.002

Data are presented as median (range) or as number (percentage). The p-values of categorical variable obtained with the Chi square or Fisher's exact test and continuous variables obtained with the Mann Whitney U-test.

*LN* lupus nephritis, *ESR* erythrocyte sedimentation rate, *anti-dsDNA* anti-double-stranded DNA, *ANA* anti-nuclear antibody, *SSA* Sjögren syndrome antigen A, *SSB* Sjögren syndrome antigen B, *rRNP* ribosomal ribonucleoprotein

*Data on 21 cases were unavailable and lupus anticoagulant was not studied.

### Adverse maternal and fetal outcomes

Patients with pre-existing LN had a significantly higher frequency of preterm delivery (81.0 % vs. 46.7%, *P* = 0.02) and cesarean section (55.3% vs. 34.3%, *P* = 0.032) compared with patients with new-onset LN. All patients with pre-existing LN had at least one episode of a composite adverse fetal outcomes, and this rate was significantly higher than that in patients with new-onset LN (97.4% vs. 80.0%, *P* = 0.018). However, the rates of low birth weight, fetal distress, fetal loss, and preeclampsia were similar between the two groups. There was no significant difference in the composite adverse maternal outcomes between the two groups (60.5% vs. 40.0%, *P* = 0.10) (Table 3).

**Table 3:** Maternal and fetal outcomes in pregnant women with LN.

	Total (n = 73)	Pre-existing LN (n = 38)	New-onset LN (n = 35)	*P* value
**Fetal outcomes**				
Gestational age, weeks*	24.0 (14.0, 34.1)	29.8 (16.5, 35.4)	20.3 (9.0, 30.4)	0.02
Live-born infant's age, weeks	35.0 (31.4,36.6)	35.2 (33.0,36.5)	32.1 (30.4,37.0)	0.13
Preterm delivery (%)^#^	24/36 (66.7)	17/21 (81.0)	7/15 (46.7)	0.02
Live-born infant's weight, kg	2.0 (1.4, 2.6)	2.1 (1.5,2.7)	1.6 (1.3,2.5)	0.27
Low birth weight (%)^#^	18/36 (50.0)	11/21 (52.4)	7/15 (46.7)	0.38
Fetal distress (%)	6 (8.2)	5 (13.2)	1 (2.9)	0.20
Fetal loss (%)	37 (50.7)	17 (44.7)	20 (57.1)	0.23
**Composite fetal outcomes (%)**	65 (89.0)	37 (97.4)	28 (80.0)	0.018
**Maternal outcomes, n (%)**				
Cesarean section (%)	33 (45.2)	21 (55.3)	12 (34.3)	0.032
Preeclampsia	14 (19.2)	10 (26.3)	4 (11.4)	0.14
**Composite maternal outcomes**	37 (50.7)	23 (60.5)	14 (40.0)	0.10

Data are presented as median (range) or as number (percentage). The p-values of categorical variable obtained with the Chi square or Fisher's exact test and continuous variables obtained with the Mann Whitney U-test.

*LN* lupus nephritis

*Including the live-born infant and fetal loss.

^#^Both denominator are the total number of live-born infant.

### Association of pre-existing/new-onset kidney disease with adverse pregnancy outcomes

After adjusting for low complement C3 levels, low serum albumin levels, proteinuria, antiphospholipid antibody and anti-rRNP antibody positive during gestation, patients with pre-existing LN had a significantly higher risk of composite adverse fetal outcomes than patients with new-onset LN (ORs, 44.59; 95% CI, 1.21–1664.82; *P* = 0.039). However, adverse maternal outcomes were similar between the two groups (ORs, 1.24; 95% CI, 0.36–4.29; *P* = 0.73) (Table 4).

**Table 4:** Effect of kidney flare/new-onset disease on adverse pregnancy outcomes.

Variables	Fetal outcomes				Maternal outcomes			
	ORs	Lower	Upper	*P* value	ORs	Lower	Upper	*P* value
Pre-existing vs. New-onset LN	44.59	1.21	1644.82	0.039	1.24	0.36	4.29	0.73
C3/ median (per 1 mg/dl)	4.83	0.20	115.01	0.33	2.08	0.69	6.25	0.19
Serum albumin (per 1 g/dL)	0.83	0.66	1.05	0.13	1.03	0.93	1.15	0.52
Proteinuria (per 1 g/24 h)	1.36	0.94	1.96	0.10	1.15	0.97	1.36	0.11
Antiphospholipid antibody positivity	5.61	0.27	117.47	0.27	1.32	0.32	5.45	0.71
Anti-rRNP antibody positive	100.54	2.73	3707.35	0.012	0.40	0.12	1.31	0.129

*LN* lupus nephritis, *rRNP* ribosomal ribonucleoprotein

### Comparison of long-term renal outcome after pregnancy

**Fig. 1:**
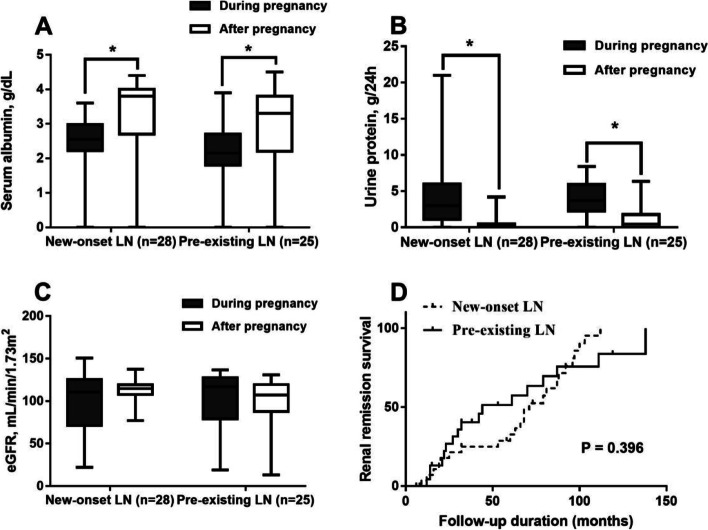
Long-term renal outcome during and after pregnancy. **A**. Serum albumin. **B**. Urine protein. **C**. eGFR. **D**. Renal remission survival the between two groups

We excluded 7 patients with dialysis therapy during pregnancy and 13 patients with lost of follow-up. During a median follow-up of 4 (1–6) years, 49 patients with 53 pregnancies (28 pregnancies were in the new-onset LN group and 25 pregnancies were in the pre-existing LN group) were included to evaluate the long-term renal outcome after pregnancy. In all patients, serum albumin levels (3.7 g/dL [3.0, 4.0] vs. 2.5 g/dL [2.1, 2.9], *P* < 0.001, Fig. 1A) and proteinuria (0.35 g/24 h [0, 0.77] vs. 3.5 g/24 h [1.42, 6.0], *P* < 0.001, Fig. 1B] among 53 pregnancies were significantly improved after pregnancy compared with those during pregnancy. However, the eGFR was not significantly different between after and during pregnancy (113.0 mL/min/1.73 m^2^ [95.4, 119.2] vs. 114.4 mL/min/1.73 m^2^ [73.0, 125.6], *P* > 0.05, Fig. 1C). A total of 12 (22.6%) pregnancies did not reach complete or partial renal remission in long-term follow-up. Kaplan–Meier analysis showed that long-term renal outcomes were not significantly different between the two groups after pregnancy (*P* = 0.396, Fig. 1D).

## Discussion

In the present study, patients with pre-existing LN were associated with a higher risk of composite adverse fetal outcomes than those with new-onset LN. However, the two groups showed similar adverse maternal outcomes. Levels of serum albumin and proteinuria significantly improved after pregnancy compared with those during pregnancy in all LN patients, and the long-term renal outcomes were not significantly different between the two groups.

Compared with patients with a history of LN, new-onset LN during pregnancy may be difficult to recognize because its signs and symptoms (including edema, proteinuria, hypertension, and renal insufficiency) may mimic glomerulonephritis and preeclampsia [18]. In our study, patients with pre-existing LN more frequently received a more aggressive treatment (prednisone plus immunosuppressant) than those with new-onset LN when nephritis was active during pregnancy. Clinicians may be more cautious about immunosuppressant therapy for pregnant patients with *de novo* LN, especially without the guidance of kidney biopsy. Our study also showed that most immunity indices were similar between the two groups, which is consistent with previous studies that compared new-onset and pre-existing SLE [10–12]. However, we found that low complement levels were more frequent in patients with new-onset LN than in those with pre-existing LN. Buyon et al. showed that low serum complement 4 levels were associated with a higher risk of developing active nephritis [18]. In fact, normal pregnancy itself is associated with systemic activation of complement [19], and the kinetics of synthesis and degradation of complement may show differences in pregnant patients with lupus.

The current study showed no significant differences in organ involvement, including blood, the heart, and the nervous system, as well as arterial hypertension, between the two groups. It is different from other studies on pregnant patients with SLE. Zhang and He et al. showed that patients with new-onset SLE suffered more hematological involvement, including thrombocytopenia and anemia, than those with pre-existing SLE [10, 11]. These differences could be explained by the fact that clinical identification of LN without kidney biopsy can be challenging because some patients often lack overt signs of extra-renal symptoms, especially in the early stage [2].

Pregnant patients with LN can be complicated by obstetric and neonatal problems, which are often concerning. In a multicenter, prospective, observational study, Moroni et al. included 71 pregnancies in 61 women with stable LN who received pre-pregnancy counseling. They showed that maternal complications developed in one third of pregnancies, and a history of renal flares before pregnancy (relative risk ratio, 10.4) predicted preeclampsia/hemolysis, elevated liver enzymes, low platelets syndrome [13]. Additionally, Chen et al. conducted a retrospective, multicenter study of 243 patients with stable SLE who underwent a planned pregnancy and close monitoring of pregnancy. In their study, 52 (21.4%) patients with disease flares mainly presented with active LN (78.8%), and disease flares (ORs, 8.1; 95% CI, 3.8–17.2) were associated with a composite of adverse fetal outcomes [8]. An observational study in Sweden included 59 pregnancies in 28 patients with SLE and showed that pre-existing LN had the strongest association for adverse pregnancy outcomes (ORs, 5.9; 95% CI, 1.7–20.8) [7].

The present study had the following distinct characteristics compared with previous studies. Firstly, different from previous studies that focused on the association of SLE or SLE flares and pregnancy outcomes [20–22], our study mainly investigated patients with LN. LN is one of the most serious manifestations of SLE and an important risk factor in mortality and morbidity. SLE with LN has a higher risk for adverse pregnancy outcomes compared with SLE without LN [23]. Secondly, even for patients with previous kidney involvement in whose nephritis is clinically quiescent at conception, LN can flare up during pregnancy. Pregnancy along with hormonal and immunological changes may affect the occurrence and disease activity of SLE [9, 24, 25], and some women could be confronted with newly-diagnosed LN. This setting is different to that in the above-mentioned studies that evaluated stable LN in patients who received pre-pregnancy counseling [8, 13]. A previous study reported that the pooled relative risk of preterm birth in patients with active nephritis was 1.78 (95% CI, 1.17–2.70) compared with those with quiescent nephritis [26]. Additionally, active proliferative LN during pregnancy was shown to be associated with small for gestational age newborns (relative risk, 3.29; 95% CI, 1.75–6.18) [27]. We found that the rates of preterm birth, low birth weight, fetal loss, and preeclampsia accounted for 32.9%, 24.7%, 50.7%, and 19.2%, respectively, in patients with active lupus, which are higher than results of previous studies [8, 13]. Therefore, controlling disease activity is essential for achieving a better outcome of LN in pregnancy.

The effect of new-onset LN on pregnancy outcomes is different from that of pre-existing LN, but relevant reports are still lacking. Our study showed that patients with pre-existing LN had more events and higher risk of composite adverse fetal outcome than patients with new-onset LN. A history of pre-existing LN is also associated with impaired renal prognosis and increased cumulative exposure of patients to drug toxic effects [28], which may increase the potential effect on fetal growth.

A total of 53.4% and 78.1% of patients with active LN experienced nephrotic proteinuria and hypoalbuminemia, respectively, during pregnancy. However, most of these patients showed significant improvement in levels of serum albumin and proteinuria after pregnancy. To the best of our knowledge, only two studies evaluated the long-term renal outcome in pregnant patients with SLE or LN [16, 29]. A retrospective cohort study showed that pregnancy did not appear to worsen renal prognosis and development of chronic kidney disease in the long term [16]. Additionally, a retrospective, nationwide, population-based cohort study showed that pregnancy was not a predictor of end-stage renal disease in pregnant patients with SLE compared with non-pregnant patients with SLE [29]. Consistent with the results of these previous studies, we observed that long-term renal outcomes improved after pregnancy and were not significantly different between the two groups.

Some limitations of our study should be acknowledged. First, this was an observational study with a relatively small sample, which introduced selection and information bias. Therefore, causality can not be determined and some residual confounding could not be eliminated. Second, because LN is a highly heterogeneous disease with ethnic differences, conclusions are only applicable to the Chinese population. Third, although some cases of new-onset LN may be hard to differentiate from preeclampsia and other glomerular diseases, some positive anti-antibodies and hypocomplementemia may represent an important feature of SLE. Fourth, only 14 patients in this cohort received kidney biopsy, which may cause early LN patients to be missed. Finally, we lacked some information on long-term outcomes of newborns and extra-renal activity of lupus of women who gave birth. However, the present study attempted to provide some beneficial information for clinicians and patients to better determine the issues of pregnancy in active LN.

## Conclusions

Pregnant patients with pre-existing LN appeared to experience more composite adverse fetal outcomes than those with new-onset LN. However, these two groups of women had a similar rate of adverse maternal outcomes. Long-term renal outcomes were not significantly different between pregnant women with pre-existing LN and those with new-onset LN. Further well-designed studies with a large sample size are required to confirm our conclusions.

## Supplementary Information

**ESM 1.**

## Data Availability

The datasets used and/or analysed during the current study are available from the corresponding author on reasonable request.

## Abbreviations

LN: lupus nephritis
SLE: systemic lupus erythematosus
eGFR: estimated glomerular filtration rate
ESR: erythrocyte sedimentation rate
anti-dsDNA: anti-double-stranded DNA
ANA: anti-nuclear antibody
SSA: Sjögren syndrome antigen A
SSB: Sjögren syndrome antigen B
rRNP: ribosomal ribonucleoprotein
