# Supplementary material for: Fetal and maternal outcome in patients with active lupus nephritis: comparison between new-onset and pre-existing lupus nephritis
Source: BMC Nephrol. 2021 Dec 21;22:419. doi: 10.1186/s12882-021-02633-2 (PMC8691084; doi:10.1186/s12882-021-02633-2)
Supplement: Supplementary file 1 — ESM 1. [file 12882_2021_2633_MOESM1_ESM.doc]

**Fetal and Maternal Outcome in Patients with Active Lupus Nephritis: Comparison between New-onset and Pre-existing Lupus Nephritis**

**Table of contents**

|  |  | **Page** |
| --- | --- | --- |
| Supplemental Fig. S1 | Flow chart | 2 |
| Supplemental Table S1 | Organ involvement and complications. | 3 |

**Fig. S1** Flow chart

Pregnant women with LN and aged ≥ 18 years (n = 77)

Excluded (n = 8)

Not active LN (n = 5);

Not a singleton intrauterine pregnancy confirmed by ultrasound (n = 0);

Drug-induced SLE and malignant tumor (n = 0 );

Absence of data of pregnancy and delivery (n = 3).

Finally included study patients

(n = 69) and pregnancies (n = 73)

Patients in new-onset group (n = 35)

Patients in pre-existing group (n = 38)

The primary endpoint: fetal and maternal adverse pregnancy outcomes (%)

**Table S1** Organ involvement and complications.

| Variables | Total  (n = 73) | Pre-existing LN (n = 38) | New-onset LN (n = 35) | *P* value |
| --- | --- | --- | --- | --- |
| **Blood system** |  |  |  |  |
| Leukopenia | 7 (9.6) | 1 (2.8) | 6 (18.8) | 0.05 |
| Anemia | 58 (79.5) | 28 (77.8) | 30 (93.8) | 0.16 |
| Thrombocytopenia | 10 (13.7) | 5 (13.9) | 5 (15.6) | 0.88 |
| **Kidney** |  |  |  |  |
| Nephrotic proteinuria | 39 (53.4) | 21 (58.3) | 18 (56.3) | 0.86 |
| Renal insufficiency | 12 (16.4) | 5 (13.2) | 7 (20.0) | 0.36 |
| Hemodialysis | 7 (9.6) | 4 (10.5) | 3 (8.6) | 0.15 |
| **Cardiovascular** |  |  |  |  |
| Pulmonary artery hypertension | 2 (2.7) | - | 2 (8.6) | - |
| Cardiac insufficiency | 4 (5.5) | 1 (2.8) | 3 (8.6) | - |
| Central nervous system | 2 (2.7) | 2 (5.3) | - | - |
| Hypertension | 16 (21.9) | 9 (23.7) | 7 (20.0) | 0.77 |
| Infection | 16 (21.9) | 7 (18.4) | 9 (25.7) | 0.45 |
| Hypoalbuminemia | 57 (78.1) | 30 (78.9) | 27 (77.1) | 0.85 |

*LN* lupus nephritis

Data are presented as number (percentage).
